# Exploring *Helianthus* Species for Resilience to Drought During the Critical Reproductive Stage

**DOI:** 10.3390/plants14040631

**Published:** 2025-02-19

**Authors:** Jelena Jocković, Nada Grahovac, Željko Milovac, Milan Jocković, Siniša Jocić, Ana Marjanović Jeromela, Sandra Cvejić

**Affiliations:** Institute of Field and Vegetable Crops, Maksima Gorkog 30, 21000 Novi Sad, Serbia; nada.grahovac@ifvcns.ns.ac.rs (N.G.); zeljko.milovac@ifvcns.ns.ac.rs (Ž.M.); sinisa.jocic@ifvcns.ns.ac.rs (S.J.); ana.jeromela@ifvcns.ns.ac.rs (A.M.J.); sandra.cvejic@ifvcns.ns.ac.rs (S.C.)

**Keywords:** wild sunflower, inflorescence, nectar, pollinators, yield

## Abstract

Drought stress during the reproductive phase of sunflower can significantly reduce achene yield by affecting inflorescence size, nectar quality, and pollinator activity. This study aimed to analyze the morphological characteristics of the reproductive region, quantify nectar sugar components, and evaluate pollinator presence and pollination success in wild *Helianthus* species as an important genetic resource for breeding cultivated sunflowers under drought conditions. Morphological investigations were conducted during the flowering and achene development phases with a stereo microscope and calipers. Nectar sugar concentrations were analyzed via HPLC, and pollinator presence was monitored twice a week for two months. This study highlights the correlation between evaluated traits, emphasizing their importance as yield indicators. Significant differences were observed in reproductive characters, nectar quality, and pollination success among the species. *Helianthus annuus* and *Helianthus argophyllus* exhibited superior reproductive performance with high nectar sugar concentrations and larger inflorescences, enabling successful pollination and higher achene yield. In contrast, *Helianthus debilis* demonstrated lower reproductive efficiency, with a higher percentage of empty achenes. These findings provide valuable insights for breeding programs, highlighting *H. argophyllus* and *H. annuus* as promising genetic resources for developing sunflower genotypes with increased yield and improved floral traits adapted to drought conditions.

## 1. Introduction

Temperature fluctuations and drought negatively impact plant growth and development, especially when these stresses occur simultaneously [1,2]. In temperate regions, the rise in temperatures and the frequency of droughts often overlap during the spring and summer months, coinciding with the reproductive phase of most field crops. This period, crucial for plant survival, makes them particularly vulnerable to stress, which can significantly disrupt plant-pollinator interactions [3,4].

Entomophilous plants, including sunflowers (*Helianthus annuus* L.), rely on the morphological characteristics of their flowers and the availability of resources such as nectar and pollen to attract pollinators and ensure successful pollination [5,6]. However, reduced water availability induces physiological modifications in plants, often leading to changes in the phenotypic traits of flowers. Research has shown that drought-stressed plants produce fewer, sparser, and/or shorter-lived flowers, as well as flowers with shorter corollas and anthers [7,8,9]. Since floral traits play a key role in plant-pollinator interactions, changes in flower size and abundance due to drought significantly affect pollination efficiency [10,11]. For example, honeybees (*Apis mellifera*), key pollinators of *H. annuus*, may have difficulties with corolla depth relative to the length of their tongue [12]. Similar behavior has been observed in bumblebees (*Bombus terrestris*), which face similar limitations. Drought-induced reductions in flower size and number lead to fewer visits by pollinator species, thereby reducing pollination efficiency and negatively impacting achene production [13].

Improving pollination efficiency, which is directly related to nectar quality, represents an important strategy for enhancing yield and sustainable food production. Nectar, as the primary resource plants offer to pollinators [14], owes its nutritional value to simple sugars: sucrose, glucose, and fructose, which are transported from the phloem to the nectary tissues [15]. In addition to the primary sugars, nectar also contains various other substances, though in much smaller amounts. These include inorganic ions, amino acids, lipids, and secondary plant metabolites, all of which contribute to the overall nutritional complexity of nectar [16]. The relationship between nectar sugar concentration and pollinator preferences has been the subject of various studies. While some suggest that sugar composition is adapted to specific pollinator groups [17], others argue that these variations are more closely related to plant phylogeny than to pollinators themselves [18]. This highlights the importance of examining both sugar composition and floral morphology, as they are not independent factors. On the contrary, nectar and floral morphological traits are interconnected elements that together function as rewards, signals, and characteristics, shaping plant-pollinator interactions and guiding future research in this field [19]. The negative impact of drought on nectar quality and quantity has been documented in previous studies [4,20]. Earlier research showed that nectar sugar concentration decreases under drought stress [21], which is attributed to reduced carbohydrate production, impaired starch transport, and phloem transport failure caused by drought [21,22]. Since pollinator attraction is directly related to nectar sugar concentration, which in turn positively affects yield, further research on genotypes that show increased nectar sugar concentration under drought conditions is a justified approach [23,24].

Drought stress not only affects nectar sugar concentration, thereby reducing pollinator attraction and indirectly impacting yield but also directly affects sunflower achene yield. Studies have shown that drought stress can lead to a decrease in sunflower seed yield by approximately 30%, with reductions in seed weight by 11% and seed numbers per head by 22% compared to normal irrigation conditions [25]. Defective fertilization due to extreme temperatures increases the percentage of empty achenes directly. Therefore, the direct consequence of empty, unfertilized seeds is a significant reduction in yield [26].

Based on the reviewed literature, the negative impact of drought on the reproductive parts of sunflowers during the flowering and seed-filling stages is evident, which consequently leads to a reduction in yield. These changes not only affect the fitness of individual plants but also have broader implications for ecosystem dynamics and pollinator interactions, particularly in the context of increased water scarcity issues due to climate change. Wild sunflower species are well-known for their remarkable drought tolerance and represent an invaluable genetic resource for improving the tolerance of cultivated sunflowers to drought conditions [27]. However, a detailed comparative characterization of the reproductive region in wild sunflower species under drought conditions has not yet been the subject of research. This research gap highlights the need for further studies to better understand how wild species can enhance plant-pollinator interactions under drought stress. Modern breeding programs are increasingly focusing on integrating adaptive traits into cultivated sunflower varieties. By utilizing the genetic diversity of wild species, it is possible to develop varieties better suited to drought-prone ecosystems, ensuring yield stability, promoting sustainable agricultural production, and making a significant contribution to global food security [27,28,29].

Therefore, the study focuses on assessing the potential of the wild annual species *H. annuus* L., *H. argophyllus* T.&G., *H. debilis* Nutt., *H. petiolaris* (A Gray) E.E., and *H. praecox* Heiser as genetic resources for improving achene yield under stress conditions. The specific objectives of the research are

1. A comparative morphological characterization of the reproductive region.
2. An analysis of the main sugar components of nectar/disc flowers.
3. Pollinator presence assessments.
4. An assessment of pollination success through yield analysis.

## 2. Results

### 2.1. Morphological Characterization of Inflorescence (Capitulum) Parts

**Figure 1:**
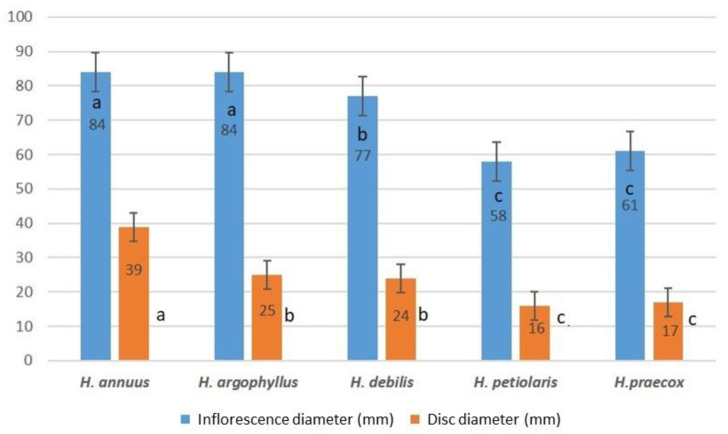
Average values of inflorescence and disk diameter in the analyzed *Helianthus* species. Duncan test: values marked with the same letter are not significantly different (the level of significance *p* ≤ 0.05).

**Figure 2:**
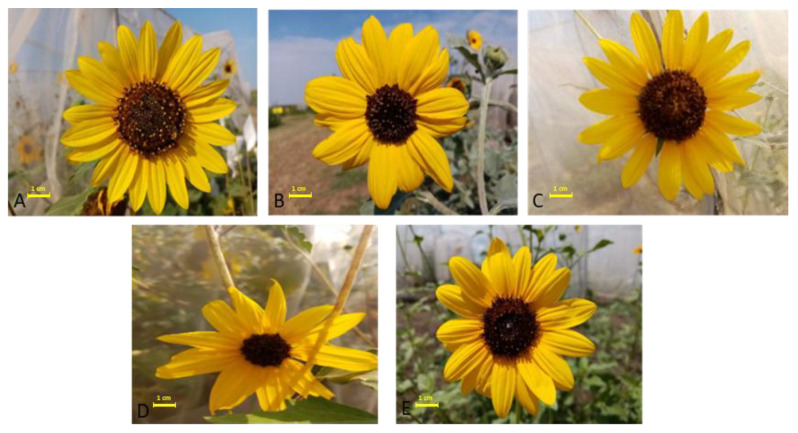
Inflorescence of the examined wild annual *Helianthus* species: *H. annuus* (**A**), *H. argophyllus* (**B**), *H. debilis* (**C**), *H. petiolaris* (**D**), *H. praecox* (**E**).

Significant species heterogeneity effects were present in the inflorescence and disc diameter (Figure 1 and Figure 2). *H. annuus* and *H. argophyllus* produce the largest inflorescence diameter (84 mm, group a), with *H. annuus* also having the largest disc diameter (39 mm, group a), while *H. argophyllus* has a smaller disc (25 mm, group b). *H. debilis* has an inflorescence diameter of 77 mm (group b) and a smaller disc diameter (24 mm, group b), placing it in the middle among all species. *H. petiolaris* (58 mm) and *H. praecox* (61 mm) have the smallest inflorescence diameters (group c) and disc diameters (16 mm and 17 mm, group c).

The results indicated significant differences in the morphological characteristics of ray flowers of five species of the *Helianthus* genus (Table 1). In all analyzed species, the ray flowers are arranged in two circles. The species *H. annuus* exhibited the highest number of ray flowers (32.2), though these were characterized by relatively shorter (30.7 mm) and narrower corollas (7.83 mm). In contrast, *H. argophyllus* had lower numbers of ray flowers (13.8) but stood out with the longest (37.4 mm) and the widest (18.53 mm) corollas. *H. debilis* displayed an intermediate number of ray flowers (19.5) with shorter corollas (27.8 mm) and moderate width (12.12 mm). *H. petiolaris* was attributed with the fewest number of ray flowers (12.3), as well as the narrowest corollas (7.70 mm). Species *H. praecox* was characterized by a smaller number of ray flowers (18) with a length of 25.4 mm and a width of 8.19 mm (Table 1).

In terms of the analyzed disc flowers, a great variability was noted among the species. *H. annuus* exhibited the longest corolla (7.45 mm) and the widest corolla in zone (1.88 mm), along with a relatively wide corolla opening (1.27 mm), as well as the longest ovary (5.35 mm). *H. argophyllus*, while having a slightly shorter corolla (7.24 mm), showed the widest corolla opening (1.48 mm) and a moderately wide corolla in the nectary zone (1.75 mm). In contrast, *H. debilis* had a shorter corolla (5.52 mm), with a smaller corolla opening (1.10 mm) and a narrower corolla in the nectary zone (1.47 mm). The corolla length of *H. petiolaris* (5.21 mm) and *H. praecox* (5.23 mm) is relatively similar; however, *H. petiolaris* exhibited a slightly longer ovary compared to *H. praecox*. *H. praecox* is a species that stands out with the smallest corolla opening (1.04 mm) and the narrowest corolla in the nectary zone (1.22 mm) among all analyzed species.

**Figure 3:**
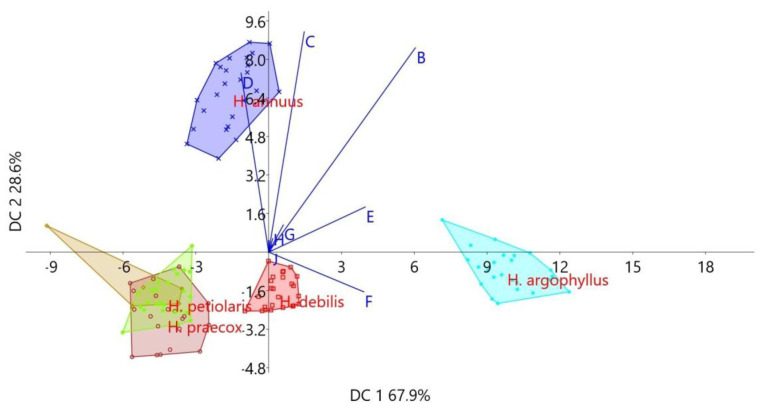
Scatter plot obtained using DCA and the position of centroids in the space of two discriminant axes, based on the inflorescence morphological characters of the studied annual *Helianthus* species.

The results of the Discriminant Component Analysis (DCA) performed on the morphological characters of inflorescences are presented in Table 2 and Figure 3. On the first discriminant axis (DC 1 67.9%), the traits that significantly contributed to the discrimination of *H. argophyllus* from the other studied species were its large disc diameter and the smallest number of ray flowers, which also exhibited the largest dimensions (both length and width) (Table 2, Figure 3). On the second and third discriminant axes, the dominant traits contributing to the species differentiation were inflorescence diameter, disc diameter, and number of ray flowers. Based on these traits, *H. annuus* clearly stands out in the positive zone of the first axis, as it exhibited the highest values for these characteristics compared to all the other analyzed species (Table 2, Figure 3).

### 2.2. Analysis of the Main Sugar Components of Nectar/Disc Flowers

**Figure 4:**
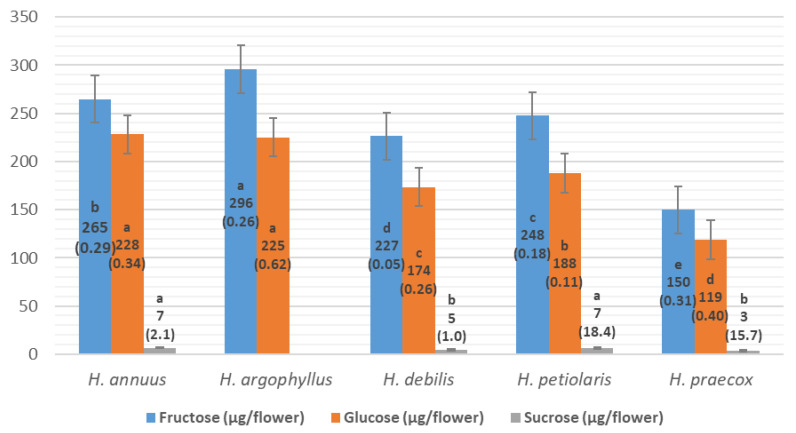
Sugar concentrations (µg) in the nectar/flowers of the studied annual *Helianthus* species (coefficient of variation CV (%)). Duncan test values marked with the same letter are not significantly different (the level of significance *p* ≤ 0.05).

**Figure 5:**
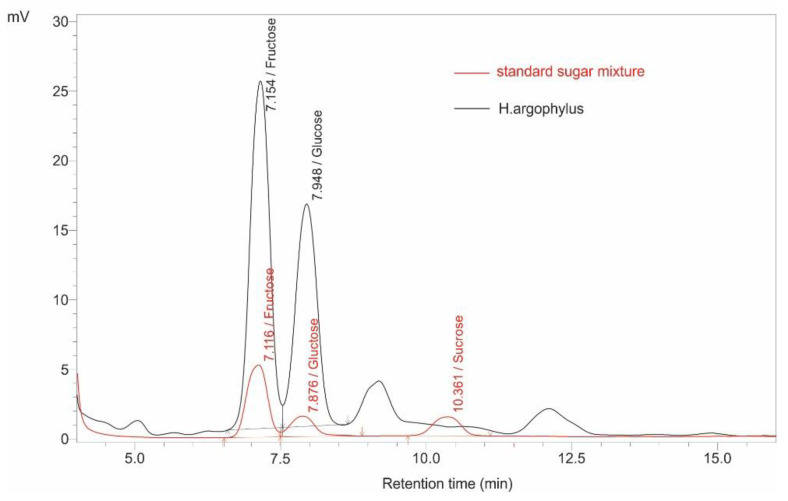
Overlaid HPLC-RID chromatograms of a standard sugar mixture (glucose, fructose, and sucrose) (red line) and the nectar/disc flower extract from *H. argophyllus* (black line). The chromatographic conditions are provided in the text.

The sugar concentrations in the nectar/flowers of the five wild sunflower species show notable differences (Figure 4). *H. argophyllus* and *H. annuus* have the highest concentrations of the investigated sugars. Specifically, *H. argophyllus* has the highest fructose concentration at 296 μg/flower and a glucose concentration of 225 μg/flower, with no detectable sucrose. In contrast, *H. annuus* produced the highest glucose concentration of 228 μg/flower, as well as the highest sucrose concentration of 7 μg/flower, with the second highest fructose concentration of 265 μg/flower. On the other hand, *H. petiolaris* (fructose: 248 μg/flower, glucose: 188 μg/flower, sucrose: 7 μg/flower) and *H. debilis* (fructose: 227 μg/flower, glucose: 174 μg/flower, sucrose: 5 μg/flower) exhibit lower sugar concentrations compared to the first two species. Finally, *H. praecox* produced the lowest concentrations overall, with fructose (150 μg/flower), glucose (119 μg/flower), and sucrose concentrations of 3 μg/flower (Figure 4). These findings suggest distinct nectar profiles among sunflower species that may influence their attractiveness to various pollinators, reflecting ecological strategies that could enhance reproductive success through targeted pollinator interactions. An example of overlaid HPLC-RID chromatograms of a standard sugar mixture (glucose, fructose, and sucrose) and a nectar/disc flower sample (*H. argophyllus*) is shown in Figure 5 (see Table 3).

Differences in nectar concentration among species are evident in their sugar and Brix values (Table 3). *H. annuus*, with a total sugar concentration of 498.7 μg/flower, has the lowest total sugars/Brix ratio (0.4), indicating that its nectar is less concentrated in sugars relative to total solids. In contrast, *H. argophyllus* (521.6 μg/flower, ratio of 0.6) and *H. petiolaris* (442.7 μg/flower, ratio of 0.8) show moderate nectar concentrations, with a higher proportion of soluble solids consisting of sugars. *H. debilis* (404.9 μg/flower, ratio of 0.9) and *H. praecox* (272.2 μg/flower, ratio of 0.8) have higher ratios, suggesting that despite their lower sugar concentrations, a greater proportion of their soluble solids consists of sugars, resulting in nectar that is relatively more concentrated in sugar compared to other total solids present.

### 2.3. Pollinator Presence Assessments

**Figure 6:**
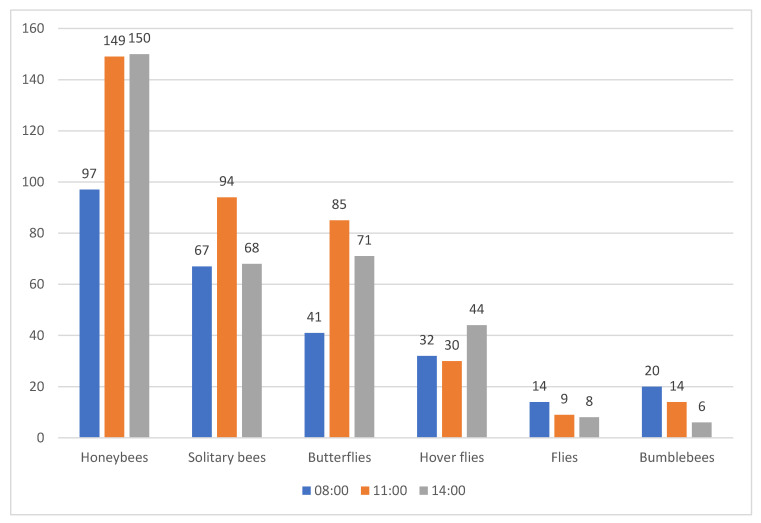
Pollinator visitations depend on the time of assessment.

Pollinator presence was assessed twice a week during three periods each day, at 08:00, 11:00, and 14:00 (Figure 6). In total, the highest pollinator abundance was recorded at 11:00—381 specimens, while at 14:00 it was 347 and at 8:00 it was 271 pollinators. Solitary bees and butterflies were most active during the second assessment, at 11:00, accompanied by a high activity of honeybees and flies were high during the first period at 8:00. Honeybee and hoverfly abundances were most dominant at 14:00. Weather conditions were unfavorable due to high temperatures and absence of rainfall. When analyzing temperatures when pollinator assessments were conducted, the average temperature for 8:00 was 24.2 °C, for 11:00 was 30.4 °C, and for 14:00 was 32.9 °C, with a clear distinction between the periods (Supplementary Figure S1).

**Figure 7:**
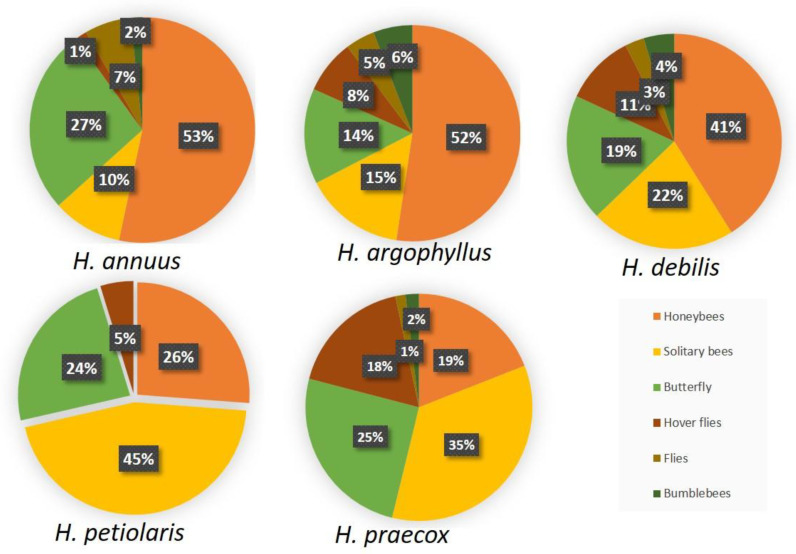
Pollinator preference estimate in percentage (%).

An analysis of pollinator preferences among different sunflower species revealed significant differences in the composition and proportions of pollinator groups, including honeybees, solitary bees, butterflies, hoverflies, flies, and bumblebees (Figure 7). *H. annuus* demonstrates a strong preference for honeybees (53%), along with contributions from butterflies (27%) and solitary bees (10%), indicating dominance of eusocial pollinators. Similarly, *H. argophyllus* is primarily visited by honeybees (52%) and solitary bees (15%), but also shows a presence of bumblebees (6%) and hoverflies (8%), suggesting broader attractiveness to various pollinator groups. In *H. debilis*, honeybees (41%) and solitary bees (22%) are prominent, while butterflies (19%) also represent a significant proportion of floral visitors, reflecting diverse pollinator attraction. Conversely, *H. petiolaris* is dominated by solitary bees (45%) compared to honeybees (26%), indicating adaptation of its flowers to smaller and less specialized pollinators. In *H. praecox*, solitary bees (35%) and butterflies (25%) are the primary visitors, while honeybee presence is reduced (19%), suggesting lower attractiveness to social pollinators and greater specialization by solitary species and lepidopterans.

### 2.4. Morphological Analysis of Achene and Examined Achene Yield Indicators

The results of the morphological analysis of the fruit (achene) and the examined yield parameters revealed statistically significant differences among the species (Table 4). The total number of achenes per head/inflorescence varied significantly, as demonstrated by Duncan’s test. *H. annuus* produced the highest number of achenes (244.5), forming a statistically distinct group (a), while *H. argophyllus* (123.8) exhibited intermediate values (b). The remaining species, *H. debilis* (50), *H. petiolaris* (81.5), and *H. praecox* (58.8), formed a separate group (c) with significantly lower achene production. Additionally, *H. annuus* had the highest percentage of full achenes (93.5%) and produced the longest (5.2 mm), the widest (2.1 mm), the thickest (1.4 mm), and the heaviest achenes (0.15 g), accompanied with the lowest percentage of empty achenes (6.5%). *H. argophyllus* had the second highest percentage of full achenes (90.9%) and slightly smaller achene dimensions (5.1 mm in length, 2.4 mm in width, 1.1 mm in thickness) with a weight of 0.10 g. The species *H. debilis* exhibited a significantly lower percentage of full achenes (58.7%) and the highest percentage of empty achenes (41.3%), with achene dimensions (5.3 mm in length, 2.0 mm in width, 1.1 mm in thickness) and weight (0.10 g) comparable to those of *H. argophyllus*. *H. petiolaris* displayed moderate values for full achenes (86.9%) and significantly smaller dimensions (3.9 mm in length, 1.6 mm in width, 0.9 mm in thickness) with a weight of 0.05 g. The smallest achenes were observed in *H. praecox*, with an average length of 3.5 mm, width of 1.3 mm, thickness of 0.9 mm, and weight of 0.04 g.

**Figure 8:**
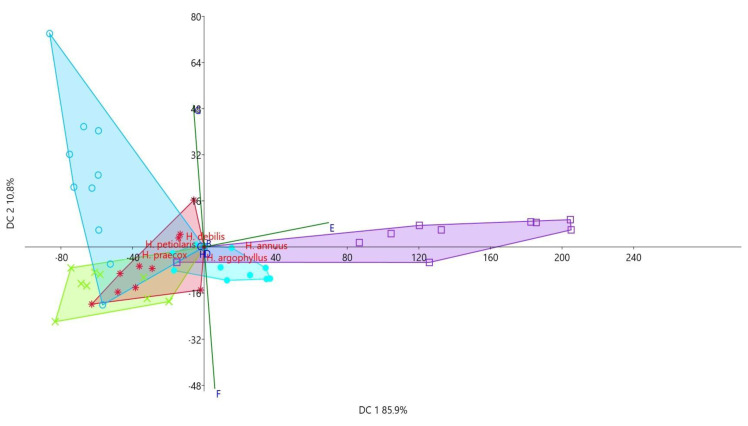
Scatter plot obtained using DCA and the position of centroids in the space of two discriminant axes, based on the analyzed yield characters of the studied annual *Helianthus* species.

The Discriminant Component Analysis (DCA) for achene traits and yield indicators revealed key differences among species (Figure 8, Table 5). On the first axis (DC 1 85.9%), the total number of achenes per head (E) was the dominant factor, with species *H. annuus* and *H. argophyllus* showing the highest values for this trait being distinctly separated. On the second axis (DC 2 10.8%), the percentage of full achenes (G) played a significant role, with the species *Helianthus debilis* notably positioned in the negative zone of the graph. Its unique placement along DC 2 is attributed to its significantly lower percentage of filled achenes. On the third axis (DC 3), achene length (B) was a key trait for species differentiation, particularly for those with larger achenes. The species *H. debilis* was characterized by the longest achenes.

**Figure 9:**
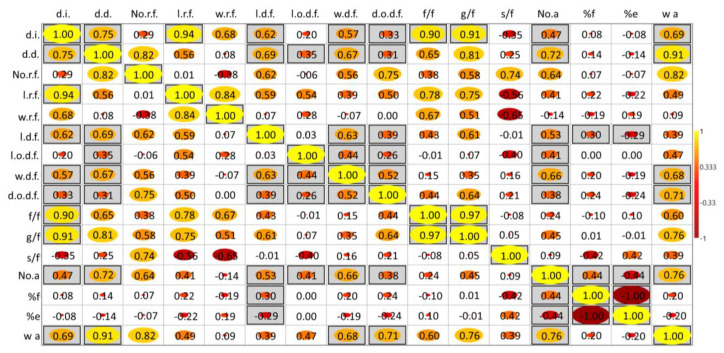
Correlation analysis (Pearson’s correlation) *p ≤ 0.05*. Coefficients in gray square are statistically significant. d.i.: inflorescence diameter, d.d.: disc diameter, No.r.f.: Number of ray flowers; l.r.f.: Corolla length of ray flower; w.r.f.: Corolla width of ray flower; l.d.f.: Corolla length of disc flower; l.o.d.f.: Length of disc flower ovary; w.d.f.: Corolla width of disc flower in nectary zone. d.o.d.f.: corolla opening width of disc flower, f/f: fructose (μg/flower), g/f: glucose (μg/flower), s/f: sucrose (μg/flower), No.a: total number of achenes per head, %f: % full achenes per head, %e: % empty achenes per head, w a: achene weight.

The correlation analyses show that inflorescence diameter (d.i.) and disc diameter (d.d.) are strongly associated with achene number (No.a) and achene weight (w a), with larger flowers leading to better achene production. Specifically, inflorescence diameter (d.i.) and disc diameter (d.d.) are linked to increased achene weight (0.69 and 0.91, respectively) and achene number (0.47 and 0.72). Ray floret traits (number, length, and width) have weaker correlations with reproductive success, while disc flower traits, such as corolla width in nectary zone (w.d.f.) and length (l.d.f.), are positively correlated with achene number (r = 0.66 and r = 0.53, respectively). Additionally, corolla opening width (d.o.d.f.) shows a correlation with glucose content (r = 0.64). Nectar sugars (fructose and glucose) are significantly interrelated (r = 0.97) and moderately correlated with achene weight (r = 0.60 for fructose, r = 0.76 for glucose) (Figure 9).

## 3. Material and Methods

### 3.1. Plant Material and Environmental Data

**Figure 10:**
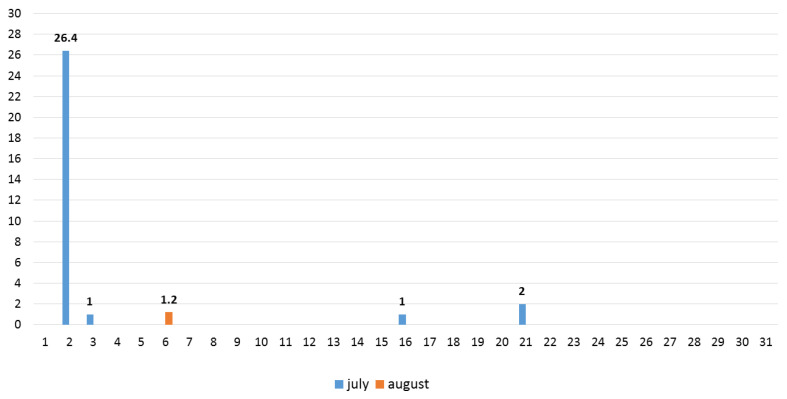
Daily precipitation (mm) during July and August of year 2024.

The study materials included five annual sunflower species: *H. annuus* L., *H. argophyllus* T.&G., *H. debilis* Nutt., *H. petiolaris* (A Gray) E.E., and *H. praecox* Heiser all observed during their full flowering phase. Since the wild *Helianthus* species have smaller achenes with thick hulls, much thicker than cultivated sunflowers, we first needed to soften the hull by placing achenes in petri dishes with distilled water for 24 h. This pre-sowing preparation started on 2 April. After that, the hull must be carefully peeled off with a scalpel and then germinated on filter paper. After radical appearance (≥1 cm), it is placed in jiffy pots (5 April). When seedlings grow approximately 5 cm they are transferred to the field, at the end of April and beginning of May (26 April–5 May). Usual agrotechnical measures are taken, without irrigation, as much as is possible in real field conditions. The size of the plot was 5 m^2^, with plant-to-plant spacing of 0.5 m and a distance between plots of 1 m. Since the primary inflorescence in branched forms of wild sunflowers completes flowering quickly, while the secondary inflorescences are more abundant and have a prolonged flowering period, the analyses in this study were focused exclusively on the secondary inflorescences due to the complexity of the research. Field experiments were conducted from early July to the end of August 2024 at the Institute of Field and Vegetable Crops, located at Rimski Šančevi in Vojvodina, Serbia. The experimental site is characterized by chernozem soil and a continental climate that ranges from semi-arid to semi-humid with pronounced drought during our observation period. In order to obtain a realistic picture of the impact of drought on the examined characters, no irrigation of the soil was carried out. July and August of 2024 in Serbia are the hottest months, when data were analyzed from 1951 to 2024, with 26 and 31 tropical days (days with average temperature ≥ 30 °C) and maximum temperatures of 39.7 °C and 40.2 °C, at the locality Rimski Šančevi [30]. Figure 10 represents daily precipitation (mm) during July and August of 2024, while the average daily temperature in the same period was 26.9 °C and 27.5 °C, respectively [30]. The average annual precipitation at the locality Rimski Šančevi, which is 647.3 mm, and the mean annual temperature of 11.9 °C [30], as well as monthly precipitations and average temperatures for July and August, are presented in Supplementary Figure S2.

### 3.2. Morphological Analysis of Inflorescence (Capitulum) Parts

The inflorescence and disk diameters of twenty selected secondary inflorescences of each species were measured using a caliper. For each inflorescence, the total number of ray flowers was recorded. Twenty-five representative, fresh ray, and disc flower samples of each examined species were randomly selected at the same developmental stage (full flowering phase). Ray flowers from the edge of the inflorescence and disc flowers from the peripheral part of the inflorescence (second open ring) were analyzed using a stereoscopic microscope STEMI 2000 (Zeiss, Oberkochen, Germany) with a KERN camera (Kern & Sohn, Balingen, Germany) and program for image analysis. The length of the corolla was measured from the base to the apex of the ray and disc flowers. Furthermore, on the same disc flowers, the ovary length was measured. The width of the corolla of the ray flowers was measured in the widest corolla part, while the width of the disc flowers was measured in the zone of the nectary and in the zone of the opening.

### 3.3. Nectar Extraction: Analysis of the Main Sugar Components of Nectar/Disc Flowers

We measured the nectar sugar concentration following flower nectar sugar washing techniques as reported by [31], with some modifications. To analyze the qualitative characteristics of nectar (dominant sugar composition: fructose, sucrose, glucose) during the flowering phase, secondary inflorescences of the analyzed species were randomly selected and isolated. Isolation was performed using special bags that allow the passage of light, water, and gases while preventing insect entry. Flowers were isolated early in the morning, at least one day before sampling the disc flowers. The next day, early in the morning, the isolation bag was removed, and six consecutive flowers from the outer ring of the inflorescence were collected from five different inflorescences and plants. Samples were directly transported to the lab to be analyzed. The fresh mass of 30 flowers was measured. The flowers were combined with 2 mL of ultra-pure water and macerated in a mortar for five minutes until the sugar was completely dissolved. The mixture was then subjected to centrifugal force for 15 min at 15,000 rpm using a Lace 16R laboratory centrifuge (Colo Lab Experts, Novo Mesto, Slovenia). The resulting supernatant was filtered through a 0.25 μm Chromafil RC-45/25 (Macherey-Nagel, Düren, Germany) syringe filter and collected in a 2 mL vial for High-Performance Liquid Chromatography (HPLC) analysis. Total sugars in the nectar/disc flowers, consisting of glucose, fructose and sucrose, were determined using the HPLC method [32]. The soluble solids residue (SSR) in the aqueous phase was determined using refractometry with a Krüss DR201-95 (A.KRÜSS Optronic, Hamburg, Germany) multi-scale digital device. The measurement unit for SSR was Brix (°Bx), calculated from the dried SSR units for a single flower (°Bx/flower) [33].

### 3.4. HPLC Analysis

The chromatographic separation and quantification of sugars (fructose, glucose, and sucrose) were performed using a Shimadzu HPLC system (LC-2050C 3D, Shimadzu Corporation, Kyoto, Japan) equipped with a refractive index detector (RID-20A, Shimadzu Corporation, Kyoto, Japan), as described by [34]. The analysis utilized an isocratic mobile phase consisting of acetonitrile (J.T. Baker, Phillipsburg, NJ, USA) and water (80:20, *v*/*v*) at a flow rate of 1 mL/min. Separation was achieved using a Nucleosil 100-5 Amino (NH_2_) column (250 × 4.6 mm, particle size 5 μm, Macherey-Nagel, Düren, Germany), with the column temperature maintained at 30 °C and the RID temperature at 40 °C. Glucose, fructose, and sucrose standards (Merck, Darmstadt, Germany) were used for sugar quantification. Glucose, fructose, and sucrose standards (Supelco, Bellefonte, PA, USA) were used for sugar quantification. A 20 μL sample volume was injected for analysis under these conditions, resulting in retention times of 6.3 min for fructose, 7.2 min for glucose, and 10.0 min for sucrose. Data acquisition and processing were carried out using LabSolutions software version 5.117 (Shimadzu Corporation, Kyoto, Japan). The sugar content was quantified and expressed as micrograms per flower (µg/flower) for each individual sugar. All measurements were performed in duplicate, and results are presented as mean ± standard deviation.

### 3.5. Pollinator Presence Assessments

Pollinator relative abundance was assessed during a period of two months (18 assessments in total, 10 plants per species), which covered most of the flowering period of all five *Helianthus* species. For this purpose, a standard visual count method was used, and every visiting pollinator insect on the secondary inflorescence was identified and classified into one of the following categories: honeybee, hoverfly, bumblebee, fly, solitary bee, or butterfly. Assessments were carried out during 5 min periods per plant species, twice a week, at 8:00, 11:00, and 14:00 in order to cover most of the pollinator activity.

### 3.6. Morphological Analysis of Achene and Examined Achene Yield Indicators

Collected achenes for morphological characterization were air-dried after collecting and stored dry at room temperature in paper bags until use. For examination of morphological characters, sixty (twenty achenes in three repetitions) randomly chosen achenes per species were selected. The size (width, length, and thickness) measurements were performed using a caliper. Length and width of each achene were measured at the longest and widest point of the achene body. The achene thickness was measured at its thickest point. The weight of twenty achenes was measured in three replicates (3 × 20) using an analytical scale. Additionally, at the technological stage of maturity, ten heads were analyzed to assess the degree of fertilization by determining the yield per head (inflorescence/capitulum). Each head was examined to determine the total number of achenes, along with the ratio of filled (full) and unfilled (empty) achenes. The results were expressed as a percentage to illustrate the proportions of filled and unfilled achenes.

### 3.7. Statistical Analysis

To assess the examined characteristics, both univariate and multivariate analyses were conducted using PAST 4.11 software. Descriptive statistics, including mean values, standard errors, correlation coefficients, and coefficients of variation, were calculated. Differences in the measured morphological parameters among the analyzed species were evaluated using Duncan’s Multiple Range Test at a significance level of *p* ≤ 0.05. Additionally, Discriminant Component Analysis (DCA) was performed to evaluate whether the analyzed sample comprised distinct groups, while correlations (Pearson’s correlation) were used to study associations between nectar quality and morphological data of analyzed reproductive parts.

## 4. Discussion

The study provided a comprehensive analysis of inflorescence morphology, nectar quality, and pollinator activity under drought conditions, focusing on the combined impact of pollination success and achene yield in wild *Helianthus* species. Monitoring sunflower inflorescence morphology serves a dual purpose: it enables an evaluation of plant attractiveness to pollinators and provides insights into factors influencing productivity and pollination success, especially under stressful conditions [35].

Inflorescence size plays a crucial role, as larger floral structures attract more pollinators, resulting in greater fruit sets and improved seed yield [36]. In line with previous research [37], the disk diameter was identified as a key indicator for high achene production in sunflowers. The species, *H. annuus* and *H. argophyllus*, with larger inflorescences and disk diameters, exhibited higher achene number and achene weight per capitulum. These results are also supported by high and positive correlation coefficients between disc diameter and achene number (0.72) and disc diameter and achene weight (0.91). Segarra [38] conducted research on cultivated sunflower genotypes in order to evaluate changes in disk diameter under drought conditions. Results indicated that drought-tolerant genotypes maintained stable disk diameters during water stress. The results of the aforementioned study supported the earlier hypothesis that drought tolerance depends on the plant’s ability to accumulate biomass in reproductive organs, such as inflorescence and achenes [39]. Our findings confirm the adaptive value of *H. annuus* and *H. argophyllus*, which demonstrate the ability to maintain inflorescence size and structure under drought conditions, ensuring reproductive success even in challenging environments.

Although ray florets significantly contribute to visual attractiveness for pollinators, our findings, consistent with earlier research [40], show that the number of ray florets is not significantly related to the percentage of full achenes per head (*p* = 0.07). Furthermore, our study demonstrated that neither the dimensions of ray florets (length and width) have a significant effect on achene yield formation, as confirmed by low coefficients of correlation. The species *H. debilis*, despite having relatively large inflorescences, is attributed with morphological traits such as smaller disc flower corolla and lower nectar sugar concentration, which may limit its attractiveness to pollinators and thus limit reproductive success. Despite its high total sugars-to-Brix ratio (0.9), indicating a significant proportion of sugars in its total soluble solids, the overall low nectar sugar concentration may still hinder its appeal to pollinators. The smaller disc flower corolla (length and width in nectary/opening zone), similar to that of *H. praecox*, could hinder nectar access for certain pollinators, potentially reducing pollination efficiency. These factors highlight that the combination of inflorescence morphology and nectar composition is crucial for reproductive success. Specifically, *H. debilis* showed the lowest percentage of full achenes (58.7%), indicating that while its large inflorescence size may attract pollinators, a lower concentration of nectar sugars or small disc flower corolla size (length, width in nectary/opening zone) may significantly reduce its reproductive output, especially important in stressful conditions such as drought.

Morphological characteristics of flowers, such as the “throat diameter” (opening for nectar access), significantly influence pollinator attraction [41]. Similar studies, such as those for the species *H. argophyllus* and *H. annuus*, have shown that species with wider and longer corollas of disc flowers are more attractive to pollinators, particularly honeybees, which require larger openings for efficient nectar collection [42]. Anatomical analyses of nectaries in perennial wild sunflower species have confirmed a positive correlation between nectary size and corolla length of disc flowers, highlighting the importance of these characteristics in attracting pollinators and their role in the reproductive efficiency of plants [35]. Our results align with these findings, showing a significant positive correlation (*p* = 0.63) between the corolla width of disc flowers in the nectary zone and corolla length, as well as with the total number of achenes per head (*p* = 0.66) and their mass (*p* = 0.68). These relationships indicate that the morphological characteristics of disc flowers play a key role in optimizing pollination efficiency and ensuring plant productivity, even under drought conditions. Furthermore, the superior reproductive efficiency of *H. annuus*, with a high percentage of fully developed achenes (93.5%) and the heaviest achenes (0.15 g), confirms that its morphological and nectar traits play a crucial role in achieving high achene yields, even in drought conditions. *H. argophyllus*, although slightly less productive, also achieved a high percentage of fully developed achenes (90.9%), further confirming the importance of flower morphology in promoting pollination success and achene formation in challenging environmental conditions.

The previous study [43] highlights the negative effect of drought on nectar resources and pollinator visits. Research on Asteraceae plants reveals that pollinators, including solitary bees, prefer flowers where their proboscises can easily reach the nectar [44]. Solitary bees have shorter proboscises compared to larger pollinators like honeybees. This anatomical trait makes them more efficient at accessing nectar from flowers with smaller corolla openings, where their size allows easier navigation and extraction of nectar [5]. These findings provide additional context for understanding the interactions observed in this study, where species like *H. debilis*, *H. petiolaris*, and *H. praecox*, with smaller corolla openings, may have been more accessible to solitary bees despite their lower nectar sugar concentrations. Despite having relatively low nectar sugar levels, the intermediate to higher total sugars/Brix ratios of *H. petiolaris* (0.8) and *H. praecox* (0.8) indicate a moderate concentration of soluble solids, which may still provide sufficient rewards for pollinators. This suggests that flower morphology, particularly corolla size, plays a crucial role in determining pollinator access, possibly compensating for lower nectar sugar concentrations.

Honeybees (*Apis mellifera* L.) are the most numerous and important pollinators in sunflower hybrid seed production, often displaying distinct preferences for specific sunflower genotypes [45]. These preferences are influenced by traits such as floret size and color, with bees using visual and olfactory cues as indicators of floral reward. Pollinators recognize various flower traits that influence their choice, with honeybees showing a preference for larger flowers over smaller ones [42]. This aligns with our observations that species like *H. argophyllus*, *H. annuus*, and *H. debilis*, characterized by larger inflorescences and disc flowers, attract the highest percentage of honeybees. Honeybee activity was most pronounced during the warmer parts of the day, particularly between 11:00 a.m. and 2:00 p.m., when high temperatures prevailed. This preference aligns with their adaptation to warmer environments and explains their dominance as pollinators during these hours [46,47]. Given that honeybees are most active during this period, they likely make the most significant contribution to the pollination of sunflower species, especially those with larger flowers and higher nectar content, such as *H. annuus* and *H. argophyllus*. Bumblebees, which are less tolerant of high temperatures, showed the highest activity early in the morning, around 8:00 a.m., which is consistent with their known preference for cooler conditions [48,49]. However, their overall abundance was significantly reduced, likely due to unfavorable environmental conditions that limit their activity. Butterflies, despite being ectothermic, were most active at 11:00 a.m. and 2:00 p.m., indicating their adaptability to heat and the reduced availability of other floral sources. Solitary bees and hoverflies were not studied as separate species but as a group due to identification challenges, but their contribution to pollination highlights the importance of maintaining diverse landscape structures and minimizing pesticide use to support a variety of pollinators [50].

## 5. Conclusions

Drought periods during the flowering stage of sunflower (*Helianthus annuus*) can significantly disrupt reproductive success through several mechanisms that affect floral structures, ultimately leading to reduced achene yield. Morphological analysis of inflorescence and analysis of nectar indicate that the yield of the investigated sunflower species under drought conditions is correlated not only with the size and the dimensions of disc flowers involved in fertilization but also with nectar quality. Together, these traits significantly influence pollination success and, consequently, achene yield. Notably, the drought-adapted species, *Helianthus annuus* and *Helianthus argophyllus*, exhibited superior reproductive performance under extremely dry conditions, as evidenced by their high nectar sugar concentrations and larger inflorescence/disc flower sizes. These traits allowed them to maintain high levels of pollination success and achieve significant achene yield, making them promising candidates for breeding programs aimed at developing drought-tolerant cultivated sunflower genotypes. Specifically, *H. annuus* and *H. argophyllus* stood out with a higher total number of achene per head and percentages of full achenes, while *H. debilis* had a significantly higher percentage of empty achenes (41.3%), underscoring its comparatively lower reproductive efficiency. Furthermore, the research underscores the essential role of pollinators, particularly honeybees, which demonstrated the highest activity during the hottest parts of the day. These pollinators provided critical pollination services, ensuring reproductive success even under stressful environmental conditions such as drought and high temperatures. Conservation efforts should therefore prioritize the protection and promotion of key pollinators, not only honeybees but also solitary bees and hover flies, since their activity directly influences sunflower yield and sustainability. This approach could significantly enhance the resilience and productivity of sunflower as a crop in the context of a challenging environment with increased frequency of drought periods and extremely high temperatures.

Also, the conclusions of this research suggest that research of this type should represent an important part of breeding programs, not only on sunflowers but also on other pollinator-dependent agricultural plants, because, with regard to the extreme climatic conditions that have occurred in recent years, crop productivity will depend not only on genetic potential but also on a number of other factors on which the degree of fertilization depends, as an important precondition for yield.

## Figures and Tables

**Table 1:** Morphological characterization of the reproductive region parts in wild annual *Helianthus* species (mean value ± standard error, coefficient of variation CV (%).

Species	No. Ray Flowers	Corolla Length of r.f. (mm)	Corolla Width of r.f. (mm)	Length d.f. with Ovary (mm)	Length d.f. Ovary (mm)	Corolla Length of d.f. (mm)	Corolla Width of d.f. in Nectary Zone (mm)	Corolla Openning Width of d.f. (mm)
*H. annuus*	32.2 ± 0.7 ^a^ (9.5)	30.7 ± 0.5 ^b^ (7.6)	7.83 ± 3.82 ^c^ (24.95)	12.78 ± 0.48 ^a^ (5.45)	5.35 ± 0.3 (25.9)	7.45 ± 1.23 ^a^ (14.88)	1.88 ± 0.06 ^a^ (12.93)	1.27 ± 0.08 ^b^ (21.92)
*H. argophyllus*	13.8 ± 0.2 ^c^ (8.0)	37.4 ± 0.3 ^a^ (3.7)	18.53 ± 2.02 ^a^ (7.67)	12.04 ± 0.12 ^b^ (2.88)	4.78 ± 0.08 (8.6)	7.24 ± 0.20 ^b^ (6.20)	1.75 ± 0.01 ^b^ (7.20)	1.48 ± 0.03 ^a^ (11.71)
*H. debilis*	19.5 ± 0.2 ^b^ (4.9)	27.8 ± 0.3 ^c^ (6.3)	12.12 ± 0.22 ^b^ (3.88)	10.24 ± 0.19 ^c^ (4.27)	4.71 ± 0.12 (8.9)	5.52 ± 0.09 c (5.55)	1.47 ± 0.05 ^c^ (15.98)	1.10 ± 0.02 ^c^ (11.79)
*H. petiolaris*	12.3 ± 0.33 ^d^ (12.1)	25.9 ± 0.3 ^d^ (6.2)	7.70 ± 0.23 ^c^ (6.29)	9.65 ± 0.28 ^d^ (5.53)	4.43 ± 0.12 (14.4)	5.21 ± 0.27 ^d^ (9.90)	1.34 ± 0.05 ^c^ (17.64)	1.17 ± 0.09 ^bc^ (23.35)
*H. praecox*	18 ± 0.5 ^b^ (12.9)	25.4 ± 0.2 ^d^ (4.7)	8.19 ± 2.43 ^c^ (19.02)	9.15 ± 0.39 ^e^ (6.81)	3.92 ± 0.08 (9.16)	5.23 ± 0.31 ^c^ (10.70)	1.22 ± 0.02 ^d^ (12.70)	1.04 ± 0.02 ^c^ (13.39)

Duncan test values marked with the same letter are not significantly different (the level of significance *p* ≤ 0.05). No.—number, r. f.—ray flowers, d. f.—disc flowers.

**Table 2:** DCA of quantitative morphological characters of inflorescence in the studied annual *Helianthus* species.

Characters	DC 1	DC 2	DC 3
Inflorescence diameter (B)	1.204	1.691	2.528
Disc diameter (C)	0.291	1.823	1.158
No. ray flowers (D)	−0.226	1.481	2.233
Corolla length of r.f. (E)	0.792	0.373	−0.541
Corolla width of r.f. (F)	0.781	−0.328	0.298
Length d.f. ovary (G)	0.120	0.224	−0.080
Corolla length of d.f. (H)	0.033	0.111	0.017
Corolla width of d.f. in nectary zone (I)	0.027	0.059	−0.003
Corolla openning width of d.f. (J)	0.025	0.015	−0.062

No.—number, r.f.—ray flowers, d.f.—dics flowers.

**Table 3:** Results of Analyzed Nectar Extracted from Flowers. (mean value ± standard error, coefficient of variation CV (%). Total Sugars (TS) represent the sum of glucose, fructose and sucrose in μg/flower.

	TS (μg/Flower)	°Brix/Flower × 10^6^	TS/°Bx
*H. annuus*	498.7 ± 1.2 ^b^ (0.3)	1150.3 ± 38.7 ^a^ (4.8)	0.4 ± 0.4 ^d^ (4.9)
*H. argophyllus*	521.6 ± 1.5 ^a^ (0.4)	885.2 ± 37.7 ^b^ (6.0)	0.6 ± 0.6 ^c^ (4.8)
*H. debilis*	404.9 ± 1.1 ^d^ (0.4)	443.3 ± 7.3 ^d^ (2.3)	0.9 ± 0.9 ^a^ (2.3)
*H. petiolaris*	442.7± 2.9 ^c^ (0.9)	576.9 ± 14.3 ^c^ (3.5)	0.8 ± 0.8 ^b^ (4.6)
*H. praecox*	272.2 ± 0.2 ^e^ (0.1)	325.1 ± 7.0 ^e^ (3.0)	0.8 ± 0.2 ^b^ (3.4)

Duncan test values marked with the same letter are not significantly different (the level of significance *p* ≤ 0.05).

**Table 4:** Achene morphological characteristics of wild annual *Helianthus* species (mean value ± standard error, coefficient of variation CV (%).

Species	Total No. of Achenes per Head	% Full Achenes Per Head	% Empty Achene per Head	Achene Length (mm)	Achene Width (mm)	Achene Thickness (mm)	Achene Weight (g)
*H. annuus*	244.5 ± 21.3 ^a^ (27.5)	93.5 ± 1.2 ^a^ (4.0)	6.5 ± 1.2 ^b^ (5.9)	5.2 ± 0.03 ^b^ (4.5)	2.1 ± 0.03 ^b^ (10.6)	1.4 ± 0.01 ^a^ (9.0)	0.15 ± 0.0008 ^a^ (9.9)
*H. argophyllus*	123.8 ± 6.4 ^b^ (16.3)	90.9 ± 1.4 ^a^ (4.8)	9 ± 1.4 ^b^ (48.9)	5.1 ± 0.04 ^b^ (6.7)	2.4 ± 0.02 ^a^ (9.03)	1.1 ± 0.01 ^b^ (13.3)	0.10 ± 0.006 ^b^ (11.2)
*H. debilis*	50 ± 2.5 ^c^ (16)	58.7 ± 8.3 ^b^ (44.9)	41.3 ± 8.3 ^a^ (63.7)	5.3 ± 0.05 ^a^ (7.4)	2 ± 0.03 ^c^ (9.8)	1.1 ± 0.01 ^b^ (13.2)	0.10 ± 0.005 ^b^ (10)
*H. petiolaris*	81.5 ± 6.6 ^c^ (25.8)	86.9 ± 2.1 ^a^ (7.8)	12.9 ± 2.2 ^b^ (53.1)	3.9 ± 0.096 ^c^ (12.5)	1.6 ± 0.02 ^d^ (13.3)	0.9 ± 0.01 ^c^ (13.1)	0.05 ± 0.0005 ^c^ (12.2)
*H. praecox*	58.8 ± 7.3 ^c^ (39.2)	90.7 ± 1.5 ^a^ (5.4)	9.2 ± 1.5 ^b^ (52.8)	3.5 ± 0.03 ^d^ (8.6)	1.3 ± 0.01 ^e^ (11.7)	0.9 ± 0.009 ^c^ (8.03)	0.04 ± 0.005 ^c^ (5.2)

Duncan test values marked with the same letter are not significantly different (the level of significance *p* ≤ 0.05; No.—number).

**Table 5:** DCA of quantitative yield characters in the studied annual *Helianthus* species.

Characters	DC 1	DC 2	DC 3
Achene length (B)	0.004	0.020	0.908
Achene width (C)	0.002	0.007	0.394
Achene thickness (D)	0.001	0.002	0.140
Total number of achenes per head (E)	0.992	0.120	−0.007
% full achenes per head (F)	0.085	−0.701	0.015
% empty achene per head (G)	−0.085	0.602	−0.014
Achene weight (E)	0.000	0.000	0.001

## Data Availability

Original data is contained within the article/Supplementary Material; further inquiries can be directed to corresponding authors as it is a result of ongoing projects.

## Supplementary Materials

The following supporting information can be downloaded at: https://www.mdpi.com/article/10.3390/plants14040631/s1, Figure S1. Temperatures on days when pollinators presence assessments were done. Figure S2. Average temperatures (a) and average precipitations (b) at the locality Rimski Šančevi, Serbia.
