# Supplementary material for: Exploring Helianthus Species for Resilience to Drought During the Critical Reproductive Stage
Source: Plants (Basel). 2025 Feb 19;14(4):631. doi: 10.3390/plants14040631 (PMC11859588; doi:10.3390/plants14040631)
Supplement: Supplementary file 1 [file plants-14-00631-s001.zip › plants-3436970-supplementary.pdf]

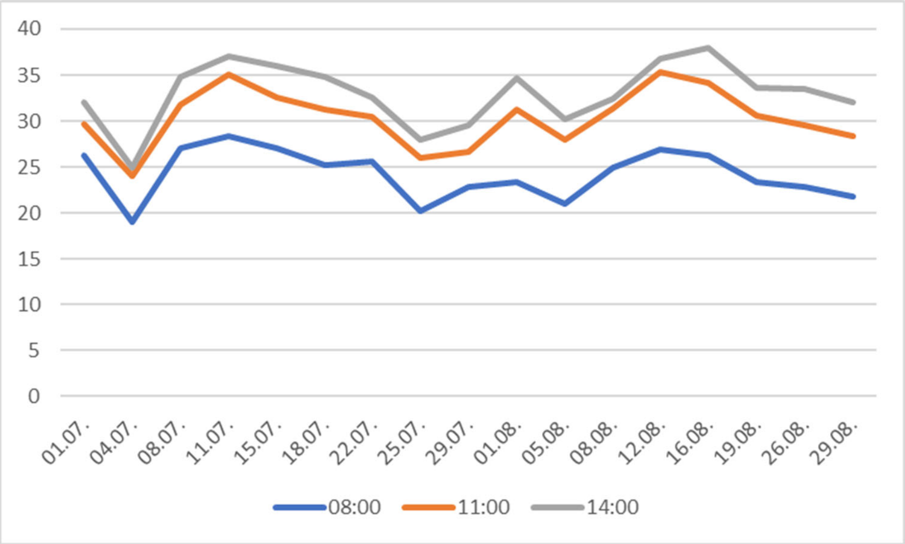

Figure S1. Temperatures on days when pollinators presence assessments were done.

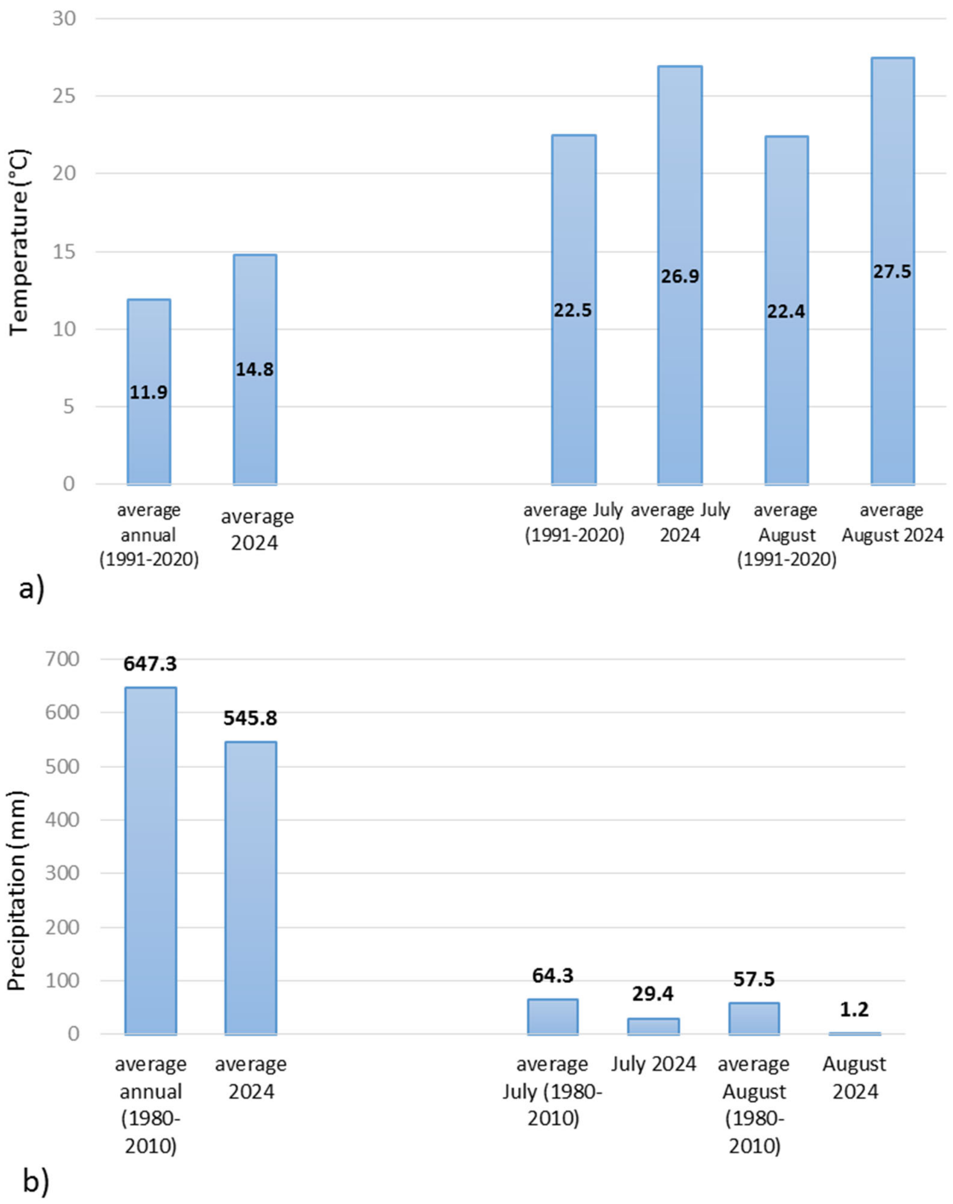

Figure S2. Average temperatures (a) and average precipitations (b) at the locality Rimski Šančevi, Serbia.
